# The Relationship of Dairy Farm Eco-Efficiency with Intensification and Self-Sufficiency. Evidence from the French Dairy Sector Using Life Cycle Analysis, Data Envelopment Analysis and Partial Least Squares Structural Equation Modelling

**DOI:** 10.1371/journal.pone.0166445

**Published:** 2016-11-10

**Authors:** Andreas Diomedes Soteriades, Alistair William Stott, Sindy Moreau, Thierry Charroin, Melanie Blanchard, Jiayi Liu, Philippe Faverdin

**Affiliations:** 1 Scotland's Rural College, Future Farming Systems Group, Edinburgh, United Kingdom; 2 Institut de l’Elevage, F-75000 Paris, France; 3 CIRAD – INRA – Montpellier SupAgro, UMR 0868 SEMLET Systèmes d'Elevage Méditerranéens et Tropicaux, F-34000, Montpellier, France; 4 Biomathematics and Statistics Scotland, Edinburgh, United Kingdom; 5 INRA, UMR 1348 PEGASE, F-35590 St-Gilles, France; 6 Agrocampus-Ouest, UMR 1348 PEGASE, F-35000 Rennes, France; University of Reading, UNITED KINGDOM

## Abstract

We aimed at quantifying the extent to which agricultural management practices linked to animal production and land use affect environmental outcomes at a larger scale. Two practices closely linked to farm environmental performance at a larger scale are farming intensity, often resulting in greater off-farm environmental impacts (land, non-renewable energy use etc.) associated with the production of imported inputs (e.g. concentrates, fertilizer); and the degree of self-sufficiency, i.e. the farm’s capacity to produce goods from its own resources, with higher control over nutrient recycling and thus minimization of losses to the environment, often resulting in greater on-farm impacts (eutrophication, acidification etc.). We explored the relationship of these practices with farm environmental performance for 185 French specialized dairy farms. We used Partial Least Squares Structural Equation Modelling to build, and relate, latent variables of environmental performance, intensification and self-sufficiency. Proxy indicators reflected the latent variables for intensification (milk yield/cow, use of maize silage etc.) and self-sufficiency (home-grown feed/total feed use, on-farm energy/total energy use etc.). Environmental performance was represented by an aggregate ‘eco-efficiency’ score per farm derived from a Data Envelopment Analysis model fed with LCA and farm output data. The dataset was split into two spatially heterogeneous (bio-physical conditions, production patterns) regions. For both regions, eco-efficiency was significantly negatively related with milk yield/cow and the use of maize silage and imported concentrates. However, these results might not necessarily hold for intensive yet more self-sufficient farms. This requires further investigation with latent variables for intensification and self-sufficiency that do not largely overlap- a modelling challenge that occurred here. We conclude that the environmental ‘sustainability’ of intensive dairy farming depends on particular farming systems and circumstances, although we note that more self-sufficient farms may be preferable when they may benefit from relatively low land prices and agri-environment schemes aimed at maintaining grasslands.

## Introduction

Meeting the world’s rapidly growing food demands in perpetuity while preserving the environment and the planet’s natural resources is an enormous challenge for agriculture [1]. The European Union (EU) is attempting to address this challenge through the Common Agricultural Policy (CAP), whose earlier focus on market price support and recent shift to direct payments and abolition of milk quotas have resulted in an intensification of dairy farming in the past few decades [2,3]. However, several dairy studies find that a trade-off might exist between dairy farm intensification and environmental performance [4–7] because intensification often has externalities associated with the production of imported inputs.

Assessing the relationship between intensification and environmental performance requires ‘global’ metrics able to capture both on and off-farm environmental impacts of dairy farming. Global metrics can be calculated with Life Cycle Analysis (LCA), an internationally standardized method for estimating the environmental impacts of agricultural products from a whole-system perspective [8]. Numerous studies use LCA to assess dairy farm environmental performance by calculating ‘eco-efficiency’ ratios, that is, environmental impacts expressed per unit of milk or land area [5,6,8,9]. Yet eco-efficiency ratios have several drawbacks [10], for example the allocation of environmental impacts to several dairy farm products (milk, meat, crops) is challenging. Dairy studies are therefore increasingly coupling LCA indicators with the multiple-input, multiple-output method Data Envelopment Analysis (DEA [11]) to calculate single aggregated eco-efficiency scores per farm, by accounting for all LCA impacts (or carbon foot-printing indicators), inputs and outputs simultaneously [10,12–19].

With global eco-efficiency indicators in hand, the next challenge is to identify farm management strategies that can improve eco-efficiency. The global efficiency indicator is based on a ratio between productions of goods and inputs (or environmental impacts). A wealth of dairy studies [5,6,8,10,13,19–22] show that eco-efficiency is generally influenced by two management strategies improving this ratio with an increase in outputs or a decrease in inputs: (i) the level of intensification at animal and farm-levels, i.e. higher production per unit of input so that increases in outputs outweigh potential increases in inputs and environmental impacts; and (ii) the farm’s degree of self-sufficiency, i.e. its capacity to produce goods from its own resources [21], so that decreases in inputs and environmental impacts outweigh likely decreases in outputs. In fact, self-sufficiency, with higher control over nutrient recycling and thus minimization of losses to the environment, can be considered as a key agro-ecological principle, central to improving the sustainability of livestock systems [23].

Nevertheless, so far a few dairy studies have attempted to holistically measure self-sufficiency, production intensity and environmental performance and to study their relationships from a whole-system perspective [21]. Certainly, this reflects the challenge of obtaining sufficient data on all three aspects [21]. Moreover, specific relationships between these aspects cannot be predetermined; it is a managerial choice to intensify production while being less or more self-sufficient, with implications for the environment in either case. On the one hand, intensive and less self-sufficient systems may increase production in a very resource use-efficient manner. For example, increasing cow productivity and the use of concentrated feeds can increase feed efficiency and milk yield per cow and per ha (10^4^ m^2^) on-farm land, and can decrease manure volume and nutrients [5,19,24]. Nonetheless, high efficiency may not be enough to reach environmental targets [25]. On the other hand, intensive and more self-sufficient systems recycle more elements on-farm, with lower environmental impacts per ha but not necessarily per unit of milk [5].

By coupling LCA data with DEA, Soteriades et al. [10] found that the less the proportion of maize silage in the total forage area of French specialized dairy farms, the higher the farms’ eco-efficiency. This confirmed earlier findings that increasing maize silage in dairy farms generally implies higher animal and farm-level farming intensity and lower farm self-sufficiency (higher supplementation with protein-rich feeds, usually imported soybean meal) [5,8,26,27]. They also showed that eco-efficiency performance can be region-dependent. Their findings suggested a possible relationship between dairy farm eco-efficiency, intensification and self-sufficiency, which could differ between regions, although, as commented earlier, these relationships cannot be predetermined. Additionally, a more holistic analysis of this relationship should involve more indicators representing these three aspects. From a modelling perspective, such cases can be dealt with the multivariate method Partial Least Squares Structural Equation Modelling (PLS-SEM [28,29]). PLS-SEM can account for multiple indicators to search for latent patterns in the data when there is no or only little prior knowledge on how the variables are related, while accounting for (regional) heterogeneity [30].

The objective of this paper was to explore the relationships between dairy farm eco-efficiency, self-sufficiency, and farm and animal-level intensification, by extending the LCA-based dairy farm exercise of Soteriades et al. [10] that accounted for impacts both on and off-farm. That way it was possible to determine whether or not on-farm management practices promoting self-sufficiency and/or increasing input per ha and output per unit input- especially per ha- could explain dairy farm environmental performance at a larger scale. In other words, this study attempts to quantify the extent to which agricultural management practices linked to animal production and land use affect output and environmental outcomes (see [31]). Another contribution of this study is that it accounted for spatial heterogeneity between farms, which could result in different relationships between eco-efficiency, self-sufficiency and intensification in each region. The relationships were explored with PLS-SEM. From a policy perspective, this study provides information for the identification of dairy farming systems that can mitigate dairy farm impacts while ensuring food security.

## Materials and Methods

This study builds on the dairy farm exercise by Soteriades et al. [10] for French specialized dairy farms (see above) which combined LCA with DEA to calculate dairy farm eco-efficiency. A brief description of their exercise (data, model and results) is therefore given below before turning to the PLS-SEM framework employed here to study the relationships of eco-efficiency with intensification and self-sufficiency.

### The study of Soteriades et al. [10]

#### LCA data and eco-efficiency

Soteriades et al. [10] used main dairy farming impacts quantified by LCA that is, non-renewable energy use, land use, eutrophication, acidification and global warming potential [6,32]. Note that in LCA studies non-renewable energy use and land use are typically considered as environmental impacts [5,6,8,32] representing ‘use of resources’ ([32], p.72). Use of resources can be seen both as resources to be used more efficiently *and* as environmental impacts resulting from the use of non-renewable resources, for instance CO_2_ emissions from combustion of fossil fuels [32]. That is why in this study non-renewable energy use and land use were considered as environmental impacts.

#### DEA and eco-efficiency scores for French specialized dairy farms

DEA was developed by Charnes et al. [11], originating from Farrell’s [33] work. It is a non-stochastic, non-parametric technique that benchmarks different decision-making units (DMUs) performing the same task in terms of their capacity to convert inputs into outputs. DEA calculates dimensionless and aggregated efficiency indices without requiring *a priori* assumptions on the importance of each variable for the DMUs’ performance, i.e. the variables’ weights are obtained from the data themselves. DEA constructs an efficient frontier, that is, a piece-wise linear surface over observed data points against which all DMUs are benchmarked. This frontier comprises of the best performers and the performance of all other DMUs is evaluated by deviations from the frontier line [34]. This is a fundamental difference between DEA and methods such as regression as the latter reflects ‘average’ or ‘central tendency’ behaviour [34,35].

The characteristics of DEA make it a particularly attractive tool for the calculation of aggregate eco-efficiency ‘scores’ as an alternative to multiple eco-efficiency ratios. This is easily demonstrated with the study of Soteriades et al. [10]. These authors fed a DEA model with the five aforementioned dairy farm LCA indicators (non-renewable energy use, land use, eutrophication, acidification, global warming potential) generated by, and three outputs (milk, meat and crop production) produced by, each French specialized dairy farm in the sample. The DEA model (known as the range adjusted measure of inefficiency [36], described in S1 Appendix) then calculated the ‘distance’ of each farm from the efficient frontier by determining by how much each LCA environmental impact should be reduced, and each output should be increased, for each farm to reach the frontier. These ‘distances’ are called inefficiencies, as they indicate that a farm is over-generating environmental impacts and/or under-producing outputs relatively to other farms in the sample. The DEA model averaged all inefficiencies for each farm to produce a score between 0 and 1. A score less than 1 meant that a farm was inefficient as it had to eliminate its inefficiencies to reach the frontier. A score of 1 meant that the farm was efficient, as its distance from the frontier- and thus its inefficiencies- were 0. The aggregation was done by the DEA model itself, which was able to weight and sum the inefficiencies altogether. Weighting the inefficiencies cancelled out the variables’ different measurement units (e.g. land use in ha, non-renewable energy use in 10^6^ J, milk in kg protein), making the aggregation meaningful. The DEA model calculated the weights from the data themselves and thus no subjective weighting choices were required. Finally, note that the range adjusted measure carries with it a ranking property allowing for the ranking of farms by their eco-efficiency scores (see S1 Appendix).

Using the aforementioned DEA model and LCA and output data, Soteriades et al. [10] calculated eco-efficiency scores for 185 French specialized dairy farms. Note that the eco-efficiency measure did not include operational inputs (e.g. labour, capital, on-farm electricity use etc.) and ‘undesirable’ outputs (e.g. kg CO_2_-equivalents, wastewater etc.) because the idea was to aggregate altogether the two elements used in LCA ratios: environmental impacts and outputs. In other words, Soteriades et al. [10] were concerned with the environmental impacts rather than the amount of operational inputs and undesirable outputs of DMUs (see [16], p.715). This is standard practice in dairy farm LCA exercises [5,6,8]. Because eco-efficiency measures an organization’s capability to produce goods and services with minimal environmental impacts [37], Kuosmanen and Kortelainen [37] argue that it represents a societal rather than a managerial perspective. Therefore, they argue that although inputs such as labour and capital are expenditures for the owners of the firm, these inputs represent income (wages and rents) for the society. Thus, such inputs are irrelevant to the context of an eco-efficiency indicator. See also sub-subsection ‘Choice of DEA variables’ in Soteriades et al. [10] and Jan et al. [16] p.715.

After obtaining the scores from the whole sample, Soteriades et al. [10] split the sample into two regions, West and Continental France, to examine if the differences in bio-physical conditions between them [38] would favour a specific region in terms of eco-efficiency performance. ‘West’ was defined by regions with oceanic climate (Basse-Normandie, Bretagne, Haute-Normanndie, Pays de la Loire, Poitou-Charentes) and ‘Continental’ by regions with continental climate (Alsace, Centre, Champagne-Ardennes, Franche-Comté, Lorraine, Rhône-Alpes). Farms on the West were called Oceanic Specialized Systems (OSS, *n* = 126) as opposed to Continental Specialized Systems (CSS, *n* = 59) in Continental France. The farms were also split into three feeding strategies (regardless of region), defined by the proportion of maize silage in the total forage area of each farm.

Results showed that OSS systems ranked higher, on average, than CSS in terms of eco-efficiency scores (mean ranks of the DEA scores were 84 for CSS and 97 for OSS). Moreover, farms with <10% and with 10–30% maize in the total forage area ranked, on average, higher than farms with >30% maize in the total forage area in terms of eco-efficiency scores (mean ranks of DEA scores were, respectively, 114, 99 and 78). As commented earlier, the latter results justified a more holistic analysis of the relationships between dairy farm eco-efficiency, intensification and self-sufficiency, leading to the current study. The former results warranted the inclusion of regional heterogeneity effects in the PLS-SEM exercise of this article.

### Exploring the relationship of eco-efficiency with intensification and self-sufficiency using PLS-SEM

**Fig 1:**
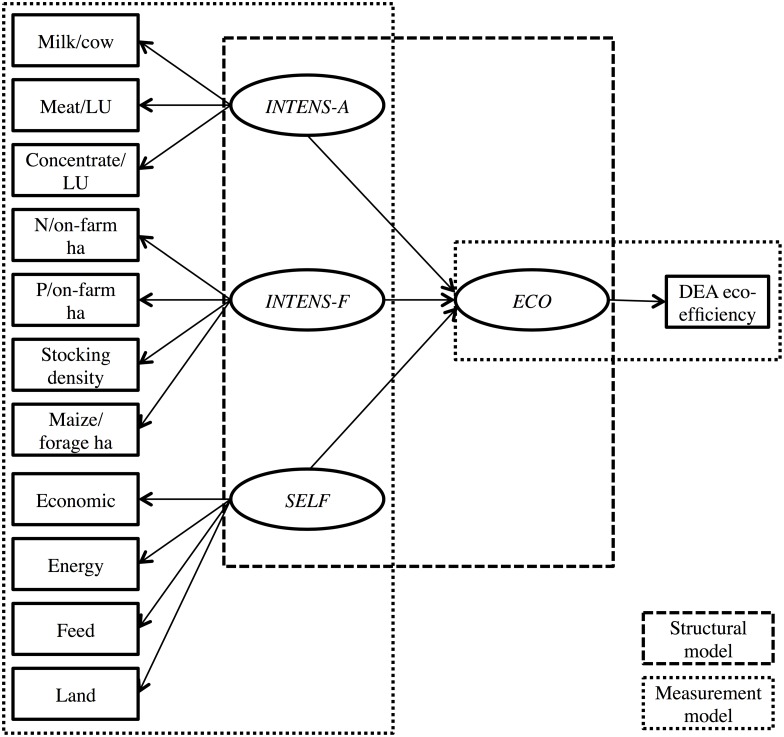
Partial Least Squares Structural Equation Model for eco-efficiency, animal and farm-level intensification, and self-sufficiency. LU: livestock unit; N: nitrogen; P: phosphorous; DEA: data envelopment analysis; *INTENS-A*: animal-level intensification; *INTENS-F*: farm-level intensification; *SELF*: self-sufficiency; *ECO*: eco-efficiency.

PLS-SEM is a structural equation modelling (SEM) approach, the latter being a general term for methods used to study the relationships among latent variables indicated by multiple manifest variables [39]. The setting of the PLS-SEM model built in the current study involved four latent variables: for eco-efficiency (*ECO*), animal-level intensification (*INTENS-A*), farm-level intensification (*INTENS-F*) and self-sufficiency (*SELF*), see Table 1 and Fig 1. *ECO* was represented by one manifest variable, the DEA eco-efficiency measure of Soteriades et al. [10]. *INTENS-A* was represented by three manifest variables, milk yield (in kg of raw milk) per cow/year (‘milk/cow’); meat production (kg carcass weight produced) per livestock unit (LU) per year (‘meat/LU’); and concentrate fed (10^3^ kg concentrate) per LU per year (‘concentrate/LU’). *INTENS-F* was represented by four manifest variables, kg mineral and organic nitrogen (N) per ha total on-farm area (‘N/on-farm ha’); kg mineral and organic phosphorous (P) per ha total on-farm area (‘P/on-farm ha’); stocking density (LU/ha main forage area); and the proportion of maize silage in the total forage area (‘maize/forage ha’).

**Table 1:** Statistics for intensification, self-sufficiency and eco-efficiency variables per system.

	CSS (*n* = 59)			OSS (*n* = 126)		
	Mean	SD	Median	Mean	SD	Median
*INTENS-A*						
Milk/cow (kg raw milk/cow/year)	6986	1560	7016	7015	1196	7095
Meat/LU (kg carcass weight produced/LU/year)	166	48	162	172	50	174
Concentrate/LU (10^3^ kg concentrate fed/LU/year)	1.00	0.41	0.98	0.95	0.38	0.98
*INTENS-F*						
N/on-farm ha (kg mineral and organic N/ha total on-farm area)	149	57	141	164	48	165
P/on-farm ha (kg mineral and organic P/ha total on-farm area)	54	23	51	60	19	57
Stocking density (LU/ha main forage area)	1.42	0.54	1.26	1.49	0.34	1.45
Maize/forage ha (% maize silage area in the total forage area)	0.20	0.20	0.20	0.28	0.16	0.30
*SELF*						
Economic (gross operating profit/turnover)	0.41	0.07	0.40	0.40	0.08	0.40
Energy (on-farm energy use/total energy use)	0.60	0.17	0.57	0.63	0.15	0.59
Feed (feedstuff produced on-farm/total use of feedstuff);	0.90	0.06	0.89	0.90	0.07	0.90
Land (on-farm land use/total land use)	0.88	0.08	0.91	0.89	0.07	0.91
*ECO*						
DEA eco-efficiency	0.94	0.05	0.93	0.95	0.05	0.95

CSS: continental specialized systems; OSS: oceanic specialized systems; LU: livestock unit; N: nitrogen; P: phosphorous; DEA: data envelopment analysis. The multiple measures building the latent constructs of animal and farm-level intensification (*INTENS-F* and *INTENS-A*) and self-sufficiency (*SELF*) were obtained from a comprehensive Life Cycle Analysis (LCA) exercise [40] resulting from a partnership involving voluntary participation of farmers (‘Inosys Réseaux d’Elevage’), the Chambers of Agriculture (France) and the French Livestock Institute. The LCA exercise is described in [10]. See same reference, text above and S1 Appendix for more details on the DEA eco-efficiency scores building the latent construct of eco-efficiency (*ECO*).

*SELF* was represented by four manifest variables of self-sufficiency: ‘economic’ (gross operating profit/turnover); ‘energy’ (on-farm energy use/total energy use); ‘feed’ (feedstuff produced on-farm/total use of feedstuff); and ‘land’ (on-farm land use/total land use). These four variables captured different dimensions of a farm’s capacity to produce goods from its own resources (i.e. to require little purchased inputs) and are complementary, providing a more holistic view of farm self-sufficiency than earlier studies (see [21]). For instance, economic self-sufficiency is an indicator of the degree of economic dependence on external inputs, e.g. pesticides (a similar indicator is used by Lebacq et al. [21]). On the other hand, land self-sufficiency indicates the extent to which a farm depends on ‘imported’ land but is unable to do so for other inputs such as pesticides and manure. Energy self-sufficiency accounts for, among others, energy required for manure for crop production, with the latter being either imported or recycled on-farm. At the same time, feed self-sufficiency alone fails to consider that farms also produce crops in addition to animal products. From this example it is evident that the four variables agree with the self-sufficiency definition adopted in this study and are complementary.

The dataset used for the PLS-SEM exercise is summarized on Table 1. See S1 File for the whole dataset. Fig 1 is a graphical representation of the PLS-SEM model built in the current study. The model is further explained below.

PLS-SEM comprises of two models (see [41]), the structural model and the measurement model (Fig 1). The structural model assumes a linear relationship between latent variables (also called constructs) and uses linear regression to estimate the path coefficients, representing the strength and direction of the relationships between the response latent variable, or target (endogenous) construct (*ECO*), and the predictor latent variables, or predictor constructs (*INTENS-A*, *INTENS-F* and *SELF*). In Fig 1 this relationship is represented by single-headed arrows from the predictor latent variables towards the response latent variable. In a similar manner, the measurement model uses linear regression to estimate the loadings, i.e. the correlations between a latent variable and its manifest variables. In Fig 1 this relationship is represented by single-headed arrows from the latent variables towards their manifest variables. The arrows’ directions imply that the constructs (*INTENS-A*, *INTENS-F*, *SELF*, *ECO*) cause the measurement (more precisely, the covariation) of their corresponding manifest variables [30]. The mathematical equations representing the PLS-SEM model in Fig 1 are presented in S2 Appendix.

PLS-SEM was deemed the appropriate SEM method to examine the relationship of *ECO* with *INTENS-A*, *INTENS-F* and *SELF* for the following reason. The procedure in PLS-SEM is an ordinary least squares regression-based estimation and thus is especially useful for developing theories in exploratory research, that is, to search for latent patterns in the data when there is no or only little prior knowledge on how the variables are related (as in this study). It does so by focusing on explaining the variance in the dependent variables (*ECO*) when examining the model, i.e. it places emphasis on prediction and theory development. This is by contrast with other SEM methods such as covariance-based SEM, which follows a maximum likelihood procedure and is thus better-suited to confirming (or rejecting) theories, i.e. it focuses on causation. See [30,42,43].

### Putting all methods together

**Fig 2:**
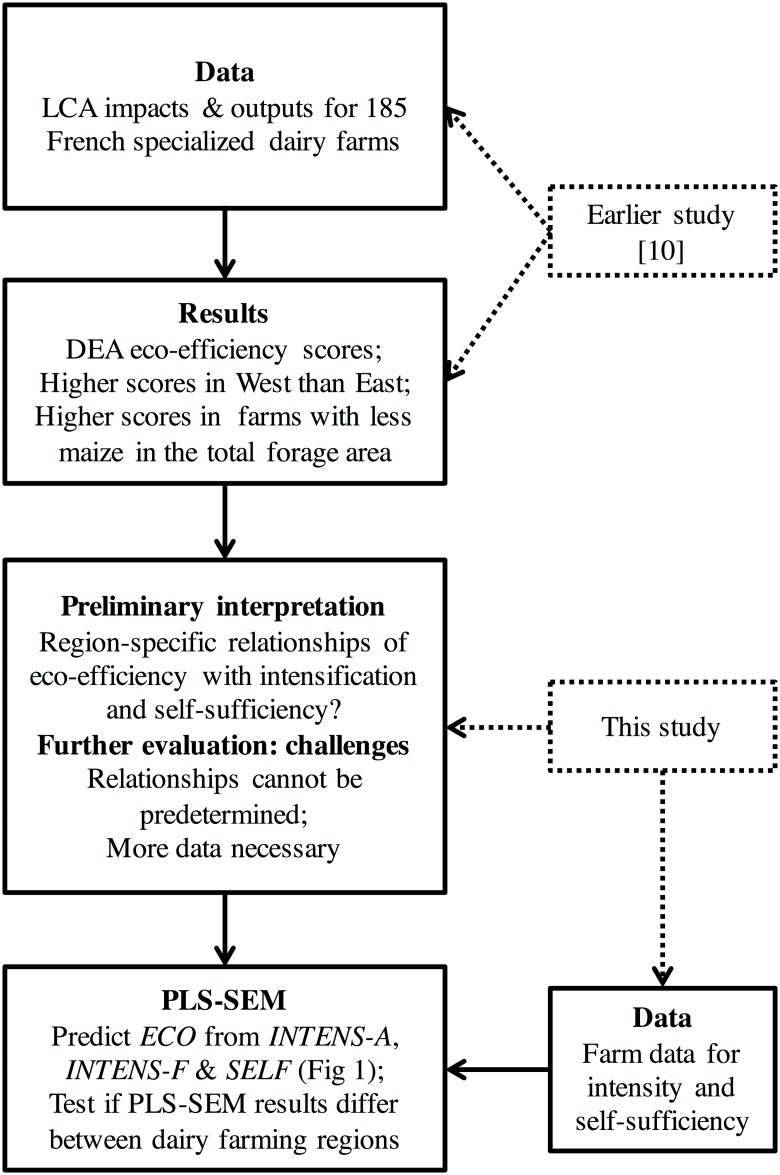
Graphical summary of how the different elements of this study’s analysis integrate. LCA: life cycle analysis; DEA: data envelopment analysis; PLS-SEM: partial least squares structural equation modelling; *INTENS-A*: animal-level intensification; *INTENS-F*: farm-level intensification; *SELF*: self-sufficiency; *ECO*: eco-efficiency.

Fig 2 summarizes graphically how the different elements of this study’s analysis integrate. Soteriades et al. [10] used LCA impacts and farm outputs to produce the DEA eco-efficiency scores for the sample of French specialized dairy farms. The results suggested a possible relationship between eco-efficiency, farming intensity and farm self-sufficiency, which could differ between regions. Further evaluation of this relationship in this study required additional data capturing several aspects of intensity and self-sufficiency. Also, modelling this relationship required a model not based on an *ad hoc* assumption of how the three elements are related, thus PLS-SEM was chosen. The eco-efficiency scores of Soteriades et al. [10] were used to build the manifest variable for *ECO* in the current study. Additional data were used in this study to create manifest variables for *INTENS-A*, *INTENS-F* and *SELF* so as to build the PLS-SEM model. Spatial heterogeneity was accounted for in the PLS-SEM model to examine if regional differences could lead to different findings in West and East France.

All calculations were performed in the R language [44]. The PLS-SEM exercise and evaluation were performed with the R package ‘plspm’ [41] and, where necessary, the first author’s own R functions.

## Results

This section summarizes the PLS-SEM results for CSS and OSS. The PLS-SEM models for both CSS and OSS complied with the minimum sample size requirements (see [30], p.20-22). Before running the exercise, the raw data were standardized (mean = 0, variance = 1) because doing so allowed the PLS-SEM model to calculate standardized coefficients between -1 and +1 for every relationship in the measurement and structural models [30].

Because PLS-SEM does not have a single goodness-of-fit criterion the measurement and structural models had to be evaluated independently and step-by-step using several assessment criteria and guidelines outlined in [30,41,42,45,46]. Given the multitude of criteria and guidelines, it is standard practice in many PLS-SEM studies (e.g. [43,47,48]) to briefly describe criteria, guidelines and their results altogether, and step-by-step. The same practice was adopted here. It should be noted that many of the criteria did not apply for single-item and/or endogenous constructs such as *ECO*, unless explicitly stated below.

The results are presented in four phases. Phase 1 is the measurement model evaluation for CSS and OSS. Phase 2 describes an issue related with the measurement model in Phase 1 and proposes alternative PLS-SEM models for CSS and OSS. Phase 3 is the measurement model evaluation of the new models. Phase 4 is the structural model evaluation of the new models.

### Phase 1: step-by-step evaluation of the measurement models for CSS and OSS

The measurement models were first evaluated for indicator reliability, which requires that the constructs should explain over 50% of their manifest variables’ variance [30]. Thus, manifest variables with loadings less than 0.70 should be removed because their construct explains less than 0.70^2^ = 0.49 ≈ 50% of their variance. The manifest variables meat/LU, P/on-farm ha, stocking density and economic and land self-sufficiency had loadings less than 0.70 for CSS. For OSS, variables with loadings less than 0.70 were meat/LU, N/on-farm ha, P/on-farm ha, stocking density and economic self-sufficiency.

Two things should be noted here. First, leaving the PLS-SEM model for OSS with just one manifest variable (maize/forage ha) representing *INTENS-F* is at odds with conventional measurement theory, according to which constructs should be typically represented by several (reflective) manifest variables (see [49], p.436). (This was not the case with *ECO* as its single manifest variable was a comprehensive measure of multiple LCA impacts and outputs. Hence, representing *ECO* by several manifest variables was unnecessary.) For that reason, N/on-farm ha, which had a loading of 0.51, was not deleted from the OSS model. Keeping manifest variables with loadings less than 0.70- but at least 0.50- is usual when situations like the present one require it (see [50], p.198). Second, the results indicated that land self-sufficiency should be retained for OSS but deleted for CSS. Retaining it in OSS would render impossible any comparisons between the PLS-SEM models for CSS and OSS as the manifest variables must be identical among models [51]. Therefore, it was removed from both models.

The final set of variables of the three exogenous constructs was, for both CSS and OSS, milk/cow, concentrate/LU, N/ on-farm ha, maize/forage ha and energy and feed self-sufficiency. The two models were then evaluated for internal consistency reliability, that is, manifest variables in the same construct should be highly correlated since they measure the same construct [30]. The criteria for internal consistency reliability used [52] were Cronbach’s *alpha* and Dillon-Goldstein’s *rho*, which should be at least 0.70, and the eigenvalues of the correlation matrix, where the first and second eigenvalues should be greater than and smaller than 1 respectively. Both models complied with these criteria, except for OSS with Cronbach’s *alpha* equal to 0.65. This value was close to 0.70 and was considered acceptable, given the aforementioned issue with N/on-farm ha but also that the criteria for Dillon-Goldstein’s *rho* and the eigenvalues were fulfilled.

The next step was to evaluate the two models in terms of convergent validity, that is, the extent to which a construct converges in its manifest variables by explaining the items’ variance [42]. Convergent validity is assessed by the average variance extracted, which equals the mean of the squared loadings for all manifest variables in a construct [42]. An average variance extracted value of 0.50 or higher is acceptable as it indicates that on average, a construct explains over 50% of the variance of its manifest variables [42]. Convergent validity was achieved in both models.

The final step was to evaluate the two models’ discriminant validity, that is, the extent to which a construct is truly distinct from other constructs in terms of how much it correlates with them, as well as how much manifest variables represent only a single construct [30]. Discriminant validity was not achieved for either model, because they failed to comply with the HTMT criterion [46]. The HTMT criterion is an estimate of the correlations between two constructs so an absolute value over 0.85 can be interpreted as a violation of discriminant validity [46,53]. All pairwise HTMT correlations between *INTENS-A*, *INTENS-F* and *SELF* had absolute values over 0.85, with their bias-corrected confidence intervals (95%, 5000 samples with replacement of farms within regions; see [30]) containing 1. The only exception was the correlation between *INTENS-A* and *INTENS-F* for OSS, with a HTMT value of 0.72 and a bias-corrected confidence interval of (0.55, 0.88). HTMT signs were positive for correlations between *INTENS-A* and *INTENS-F* and negative between *INTENS-A* and *SELF* and between *INTENS-F* and *SELF*.

The HTMT results provided strong evidence that *INTENS-A*, *INTENS-F* and *SELF* were not truly distinct from each other in either model. This required a complete reconsideration of the PLS-SEM model displayed in Fig 1.

### Phase 2: re-considering the PLS-SEM model

**Fig 3:**
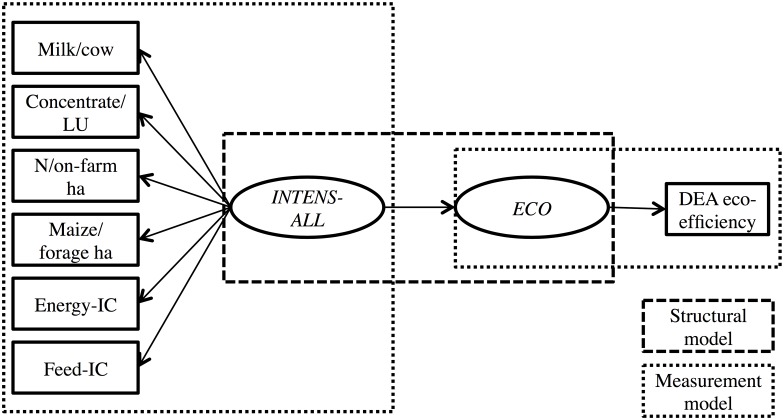
Partial Least Squares Structural Equation Model for eco-efficiency and intensification (including inverse-coded self-sufficiency variables). LU: livestock unit; IC: inverse-coded; DEA: data envelopment analysis; *INTENS-ALL*: animal and farm-level intensification, as well as inverse-coded self-sufficiency; *ECO*: eco-efficiency.

**Fig 4:**
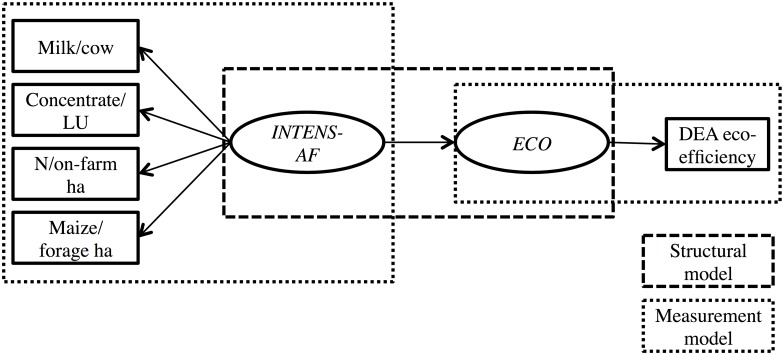
Partial Least Squares Structural Equation Model for eco-efficiency and intensification (self-sufficiency variables removed). LU: livestock unit; DEA: data envelopment analysis; *INTENS-AF*: animal and farm-level intensification; *ECO*: eco-efficiency.

The HTMT results suggested that the three exogenous latent variables *INTENS-A*, *INTENS-F* and *SELF* measured ‘the same thing’. In fact, the negative HTMT correlations of *SELF* with *INTENS-A* and *INTENS-F* suggested that *SELF* could be better suited as a measure of intensification; that is, the lower the self-sufficiency levels the higher the intensification levels. Therefore, the PLS-SEM model displayed in Fig 1 was replaced by the PLS-SEM model in Fig 3, where the manifest variables for *SELF* were inverse-coded to reflect intensification, because that way the higher the self-‘insufficiency’ levels the higher the intensification levels. (Inverse-coding is standard practice in PLS-SEM when facing similar issues. It is done by multiplying the manifest variables of interest by -1, see [30,41].) The new exogenous latent variable, comprising of *INTENS-A*, *INTENS-F* and inverse-coded *SELF* is denoted as *INTENS-ALL*. It should be made clear though that by no means can this result be generalized to suggest that more intensive farms are less self-sufficient. This result was rather specific to the available dataset and is further discussed in the Discussion section. Therefore, a second PLS-SEM model was considered, where the manifest variables for *SELF* were completely removed (Fig 4). In this case, the new exogenous latent variable, comprising of *INTENS-A* and *INTENS-F* is denoted as *INTENS-AF*. The two new PLS-SEM models were named accordingly, that is, PLS-SEM-ALL (Fig 3) and PLS-SEM-AF (Fig 4). The mathematical equations representing these two models are presented in S3 Appendix.

PLS-SEM-ALL and PLS-SEM-AF were run for CSS and OSS. The results for the new measurement models for CSS and OSS are reported in Phase 3 below.

### Phase 3: step-by-step evaluation of the new measurement models for CSS and OSS

The final set of manifest variables kept in PLS-SEM-ALL for both CSS and OSS was milk/cow, concentrate/LU, maize/forage ha, and energy and feed self-‘insufficiency’. For PLS-SEM-AF, the final set of manifest variables was milk/cow, concentrate/LU and maize/forage ha for both CSS and OSS. Skewness and kurtosis values for all aforementioned variables, as well as for DEA eco-efficiency, were between -1 and +1 and thus complied with the requirement that with PLS-SEM data can only slightly depart from normality (this minimizes the chances of obtaining unreliable results, see [30]). The only exception was milk/cow for CSS with kurtosis of -1.29, which was not ‘too far’ from -1 and was not considered a problem [30,54].

The final measurement models complied with all criteria for indicator reliability, internal consistency reliability and convergent validity (S1, S2, S3 and S4 Tables). Bootstrapped confidence intervals (95%, 5000 samples with replacement of farms within regions; see [30]) showed that all the relationships between the manifest variables and their construct were significant (S1, S2, S3 and S4 Tables).

### Phase 4: evaluating the structural models for CSS and OSS

The first step of the structural model assessment was to determine the structural models’ predictive accuracy and relevance by means of *R*^2^, *R*^2^-adjusted and Stone-Geisser’s cross-validated *Q*^2^ (blindfolding) [30].

The *R*^2^ is a measure of the proportion of the endogenous construct’s (*ECO*) variance that is explained by the exogenous constructs (*INTENS-ALL* or *INTENS-AF*). For PLS-SEM-ALL the *R*^2^ values were 0.13 (CSS) and 0.33 (OSS). The respective *R*^2^-adjusted values, that is, *R*^2^ adjusted for sample size to allow for comparisons between models, were 0.11 for CSS and 0.32 for OSS. For PLS-SEM-AF, the *R*^2^ (*R*^2^-adjusted) values were 0.09 (0.08) for CSS and 0.31 (0.30) for OSS.

The *Q*^2^ is a measure of the structural models’ predictive relevance and values above 0 are considered acceptable. For PLS-SEM-ALL *Q*^2^ ranged between 0.08 and 0.11 for CSS and between 0.29 and 0.32 for OSS, indicating, respectively, small and large predictive relevance for CSS and OSS. For PLS-SEM-AF *Q*^2^ ranged between 0.05 and 0.08 for CSS and between 0.27 and 0.29 for OSS, again indicating, respectively, small and large predictive relevance for CSS and OSS.

The second and final step was to evaluate the strength and significance of the structural models’ path coefficients. The results and their bootstrapped estimates (95%, 5000 samples with replacement of farms within regions; see [30]) are presented in Table 2. The path coefficients *INTENS-ALL* → *ECO* and *INTENS-AF* → *ECO* were significant and negative for both CSS and OSS. By means of a permutation test (5000 repetitions) [55], significant differences at *p* < 0.05 were found between the path coefficients of CSS and OSS for both PLS-SEM-ALL (*p* = 0.036) and PLS-SEM-AF (*p* = 0.024) (Table 2).

**Table 2:** Path coefficients and their bootstrapped confidence intervals, standard errors, *t*-values and *p*-values for the final structural models for CSS and OSS.

Path	Path coefficient (95% CI)	SE	*t*-value	*p*-value
*INTENS-ALL* → *ECO* (CSS)	-0.36^a^ (-0.55, -0.21)	0.124	-2.860	0.001
*INTENS-ALL* → *ECO* (OSS)	-0.57^b^ (-0.67, -0.47)	0.074	-7.770	< 0.001
*INTENS-AF* → *ECO* (CSS)	-0.31^a^ (-0.51, -0.15)	0.126	-2.416	0.019
*INTENS-AF* → *ECO* (OSS)	-0.55^b^ (-0.65, -0.45)	0.075	-7.369	< 0.001

*INTENS-ALL*: exogenous construct in model PLS-SEM-ALL, consisting of manifest variables for animal and farm-level intensification, and inverse-coded self-sufficiency; *INTENS-AF*: exogenous construct in model PLS-SEM-AF, consisting of manifest variables for animal and farm-level intensification; *ECO*: eco-efficiency, endogenous construct in both PLS-SEM-ALL and PLS-SEM-AF; CSS: continental specialized systems; OSS: oceanic specialized systems; CI: confidence interval; SE: standard error. Values within a column with different superscripts differ significantly at *p* < 0.05. Note that superscripts are not comparable between PLS-SEM-ALL and PLS-SEM-AF.

## Discussion

This paper studied the relationship between dairy farm eco-efficiency, self-sufficiency, and farm and animal-level intensification so as to quantify the extent to which agricultural management practices linked to animal production and land use affect output and environmental outcomes at a larger scale. Importantly, this paper accounted for spatial heterogeneity in dairy farms, an increasingly recognized potentially differentiating factor of dairy farm performance [10,18,56–59]. From a policy perspective, this study provides information for the identification of dairy farming systems that can help mitigate dairy farm impacts while ensuring food security.

### Eco-efficiency, intensification and self-sufficiency: relationships, overlaps and recommendations

As noted earlier, a possible confusion could arise from the HTMT results in that they suggested that the higher the (animal and farm-level) intensification, the lower the self-sufficiency. This finding resulted from the available dataset, but it is not always the case. Counterexamples include (i) the ‘average’ New Zealand dairy farm that is intensive per ha on-farm land, however imports less than 10% of total feed offered [5]; and (ii) intensive grazing systems in Belgium with high milk yields per cow and ha [22]. Both cases suggest a synergy between intensification and self-sufficiency, contrary to what was found in this study.

Nevertheless, in the case of this study’s 185 French specialized dairy farms, the negative relationship between intensification and self-sufficiency indicated by the HTMT results is fairly intuitive. Indeed, half the country’s milk production comes from more intensive systems making heavy use of maize silage (especially in the West), which requires supplementation with protein-rich complements, mainly soybean meal imported from Brazil [26,27,60], consequently reducing the farms’ self-sufficiency levels.

The heavy use of maize silage also has implications for the farms’ environmental performance. We confirmed earlier studies [5,10,20] arguing that heavy reliance on maize silage and/or concentrates can reduce dairy farm environmental performance. This is because importing soybean meal to complement maize silage-based diets of more intensive systems implies major environmental impacts at the point of production from the expansion of soy area and forest clearing [26,61]. Note that these impacts were fully accounted for in the LCA indicators used to derive the eco-efficiency scores thus revealing the true implications of on-farm management decisions for the environment.

The results also confirmed that although increasing milk yield per cow can improve environmental performance at the cow-level [8,9], this does not necessarily hold *at whole farm-level* [62,63]. The role of DEA enhanced the validity of this finding because the method does not express each impact per some functional unit (e.g. unit of milk or land), choice of which may lead to radically different conclusions [64]; DEA rather aggregates farm impacts and outputs *altogether*, providing a single eco-efficiency measure for the whole farm. It is noteworthy, however, that two recent DEA studies found that intensification at cow-level actually resulted in better environmental performance at farm-level, regardless of feed self-sufficiency levels as reflected by two different feeding practices (lower *versus* higher reliance on imported concentrates) [13,19]. These findings demonstrate the potential for intensive farms to improve environmental performance [9], perhaps by adoption of highly self-sufficient management practices, in line with of agro-ecological concepts.

The concept of agro-ecology considers agro-ecosystems as a whole in terms of several aspects (biological, technical, economic, etc.) and aims at eliminating the disconnectedness of livestock farming from the land [23]. More self-sufficient systems are more in line with agro-ecology because they can better regulate biogeochemical cycles and environmental fluxes to the atmosphere and hydrosphere through interactions among different farm units in space and time [23]. When stocking densities are kept at lower levels, adequate cropland on-farm offers a high potential for the recycling of manure-related nutrients [65]. By coupling agro-ecological concepts with other practices that reduce reliance on external nutrient sources (e.g. by improving the efficiency of nutrient utilization by the animals; [23]), self-sufficiency then has the potential for high environmental performance without necessarily lowering a farm’s intensification level [5,22] or, in some cases, by just moderately reducing productivity/ha (see RAD, 2010 in [23]). Given that in our sample more grass-based farms exhibited higher eco-efficiency than more maize silage-based farms (see [10]), intensive yet self-sufficient farms with higher reliance on grass than maize silage may then be preferable because (i) land in France is relatively cheap [66]; (ii) farms could benefit from agri-environment schemes aimed at maintaining grasslands, for example France’s ‘prime à l’herbe’ grassland support scheme [2].

In summary, contrasting results between studies leave less room for a definitive answer as to whether or not intensification is beneficial for the environment; it depends on particular farming systems and circumstances. However, increasing self-sufficiency could offer a way to improve the eco-efficiency of intensive dairy farms, in line with ago-ecological concepts and the consensus that food production should be increased with the least possible environmental impacts [67,68]. In any case, PLS-SEM is very advantageous for exploring relationships between potentially overlapping elements, especially when these relationships are theory-skeletal, which is the case with eco-efficiency, intensification and self-sufficiency. Finally, feeding PLS-SEM with data derived by LCA and DEA enhanced the credibility of the findings.

### The pros and cons of PLS-SEM to study farm sustainability

A wealth of methods has been employed to aggregate different farm sustainability and/or efficiency indicators and/or to explore the relationships between them. Methods include bivariate and multivariate statistics, linear programming, DEA, LCA, simulation, and monitoring tools [4,8,9,16,18,69–73]. In this study, PLS-SEM was deemed as the appropriate exploratory tool for two main reasons. First, PLS-SEM allows for the aggregation of manifest variables into latent variables, as opposed to simply feeding the manifest variables altogether into, for example, a regression [9] or principal component analysis [8] model. Second, with PLS-SEM it is possible to simultaneously explore the relationships between more than two latent variables at a time, contrary to bivariate statistics that can only handle pairs of variables. Actually, the first and second aforementioned advantages derive from the fact that PLS-SEM can analyse the whole model as a unit, rather than dividing it into pieces (adopted from [54]).

PLS-SEM combined with LCA and DEA has potential as a guiding tool for the identification of more sustainable dairy farming practices and the formation of environmental regulations. Recent studies argue that environmental externalities are unlikely to decrease solely as a result of market-based instruments and the free market-oriented CAP reforms, hence some policy intervention is necessary [7,74]. Consider, for example, dairy farms in West France. These farms are generally highly industrialized and competitive and have demonstrated a greater ability to respond to CAP’s shift from milk support prices to direct payments and towards trade liberalization than farms in other areas [60]. Yet, this study demonstrated that such farms might perform poorly in terms of environmental performance when considering market *and* non-market goods, as well as local and ‘imported’ impacts and spatial heterogeneity (recall results in Table 2). Hence, our holistic, ‘global’ framework could help guide the formation of local environmental regulations that typically ignore ‘imported’ impacts of farming, potentially resulting in self-sufficient farms experiencing greater enforcement as they tend to generate higher local impacts than less self-sufficient farms.

On the downside, a widely recognized problem with the assessment of PLS-SEM is that, unlike other SEM models, it does not have a standard goodness-of-fit statistic [75]. For instance, there is no universal agreement as to which value of *R*^2^ is considered acceptable and in some cases a value as low as 0.10 is satisfactory [42]. A general guideline is to interpret *R*^2^ in the context of the study at hand by considering *R*^2^ values from related studies [42]. This was impossible to do in this study for the following reason. From the six applications of SEM to dairy farming identified in the literature [47,76–80], only Gyau et al. [47] employed PLS-SEM as the preferred SEM method and the objective of their study had no relevance to that of the current study. Consequently, it was hard to draw any conclusions on the structural model’s performance based on the *R*^2^ values obtained in this study. It should be admitted though that the *R*^2^ values for CSS were probably too low and a future step would be to develop a better PLS-SEM model for CSS with more data. At least, the positive *Q*^2^ values indicated the predictive relevance of the structural models, especially for OSS where the values were ‘well above zero’ ([42], p.111). It is noteworthy that in other studies *R*^2^ and *Q*^2^ values as low as for CSS were considered acceptable (e.g. [43]). Other indices to judge the overall model fit in PLS-SEM models have been suggested, such as the global and relative goodness-of-fit indices (see [75]). However, these indices have proven unsuitable for model validation [75] and were not used here.

In summary, the advantages of PLS-SEM as an exploratory tool to study farm sustainability lie on the fact that it can analyse the whole model as a unit, rather than dividing it into pieces. When the PLS-SEM analysis is enriched by holistic LCA and DEA data, the method has potential as a guiding tool for the identification of more sustainable dairy farming practices and the formation of environmental regulations. A disadvantage of PLS-SEM is that it has no standard goodness-of-fit statistic and so its performance can only be assessed with general guidelines and by comparisons with other studies.

## Conclusions

The findings of the current study suggested that whether intensive dairy farming is ‘good’ or ‘bad’ for the environment depends on particular farming systems and circumstances. In this study, on-farm management practices such as increased reliance on maize silage and bought-in concentrates reduced eco-efficiency when the latter was assessed at a ‘global’ level with the LCA-based DEA eco-efficiency scores. The same was true for the effect of increasing milk yield/cow on eco-efficiency because other products (meat and crops) and their associated impacts were also accounted for in the aggregate DEA eco-efficiency scores. However, these results might not necessarily hold for intensive farms with higher self-sufficiency levels and thus better ability to recycle elements on-farm. Intensive yet self-sufficient farms with higher reliance on grass than maize silage may be preferable in cases where they may benefit from relatively low land prices and agri-environment schemes aimed at maintaining grasslands. Finally, our holistic, ‘global’ framework proved to be a valuable tool for quantifying the extent to which agricultural management practices linked to animal production and land use affect output and environmental outcomes, and could help guide the formation of local environmental regulations that typically ignore ‘imported’ impacts of farming.

## Supporting Information

S1 Appendix RAM: range adjusted measure. (DOCX)

S2 Appendix Equations for the PLS-SEM model in Fig 1. (DOCX)

S3 Appendix Equations for PLS-SEM-ALL and PLS-SEM-AF. (DOCX)

S1 File The dataset used in this study. CSS: continental specialized systems; OSS: oceanic specialized systems; *INTENS-A*: animal-level intensification; *INTENS-F*: farm-level intensification; *SELF*: self-sufficiency; *ECO*: eco-efficiency. Note that the data for *ECO* were derived by [10].
(XLSX)

S1 Table Indicator reliability, internal consistency reliability and convergent validity of the measurement model for PLS-SEM-ALL for Continental Specialized Systems (CSS). *INTENS-ALL*: animal and farm-level intensification, and inverse-coded self-sufficiency indicators; *ECO*: eco-efficiency; IC: inverse-coded; CI: confidence interval; AVE: average variance extracted; LU: livestock unit; DEA: data envelopment analysis; N/A: not applicable. Rules of thumb [30,42,45,52]: loadings, Cronbach’s *alpha* and Dillon-Goldstein’s *rho* should be at least 0.70. Communalities (squared loadings) and AVE should be at least 0.50. First and second eigenvalues should be above and below 1 respectively. Note: *ECO* is a single-item construct so assessment criteria above do not apply.
(DOCX)

S2 Table Indicator reliability, internal consistency reliability and convergent validity of the measurement model for PLS-SEM-ALL for Oceanic Specialized Systems (OSS). *INTENS-ALL*: animal and farm-level intensification, and inverse-coded self-sufficiency indicators; *ECO*: eco-efficiency; IC: inverse-coded; CI: confidence interval; AVE: average variance extracted; LU: livestock unit; DEA: data envelopment analysis; N/A: not applicable. Rules of thumb [30,42,45,52]: loadings, Cronbach’s *alpha* and Dillon-Goldstein’s *rho* should be at least 0.70. Communalities (squared loadings) and AVE should be at least 0.50. First and second eigenvalues should be above and below 1 respectively. Note: *ECO* is a single-item construct so assessment criteria above do not apply.
(DOCX)

S3 Table Indicator reliability, internal consistency reliability and convergent validity of the measurement model for PLS-SEM-AF for Continental Specialized Systems (CSS). *INTENS-AF*: animal and farm-level intensification; *ECO*: eco-efficiency; CI: confidence interval; AVE: average variance extracted; LU: livestock unit; DEA: data envelopment analysis; N/A: not applicable. Rules of thumb [30,42,45,52]: loadings, Cronbach’s *alpha* and Dillon-Goldstein’s *rho* should be at least 0.70. Communalities (squared loadings) and AVE should be at least 0.50. First and second eigenvalues should be above and below 1 respectively. Note: *ECO* is a single-item construct so assessment criteria above do not apply.
(DOCX)

S4 Table Indicator reliability, internal consistency reliability and convergent validity of the measurement model for PLS-SEM-AF for Oceanic Specialized Systems (OSS). *INTENS-AF*: animal and farm-level intensification; *ECO*: eco-efficiency; CI: confidence interval; AVE: average variance extracted; LU: livestock unit; DEA: data envelopment analysis; N/A: not applicable. Rules of thumb [30,42,45,52]: loadings, Cronbach’s *alpha* and Dillon-Goldstein’s *rho* should be at least 0.70. Communalities (squared loadings) and AVE should be at least 0.50. First and second eigenvalues should be above and below 1 respectively. Note: *ECO* is a single-item construct so assessment criteria above do not apply.
(DOCX)
