# Supplementary material for: The Relationship of Dairy Farm Eco-Efficiency with Intensification and Self-Sufficiency. Evidence from the French Dairy Sector Using Life Cycle Analysis, Data Envelopment Analysis and Partial Least Squares Structural Equation Modelling
Source: PLoS One. 2016 Nov 10;11(11):e0166445. doi: 10.1371/journal.pone.0166445 (PMC5104379; doi:10.1371/journal.pone.0166445)
Supplement: S1 Appendix — (DOCX) [file pone.0166445.s001.docx]

# S1 Appendix. RAM: range adjusted measure

Suppose that there are $n$ DMUs each using $m$ inputs (or environmental impacts in the case of this study) to produce $s$outputs, denoted as $x_{i}$ $\left(i=1,...,m\right)$ and $y_{r}$ $\left(r=1,...,s\right)$ respectively. The RAM inefficiency score of the *j*th DMU, denoted as DMU_o_, is given by the following linear program [1]:

$\rho^{*}=\max_{\lambda_{j},s_{io},s_{ro}}\frac{1}{m+s}\left(\sum_{i=1}^{m} \frac{s_{io}}{R_{i}}+\sum_{r=1}^{s} \frac{s_{ro}}{R_{r}}\right)$ (1)

subject to

$$\begin{matrix} x_{io}=\sum_{j=1}^{n} x_{ij}\lambda_{j}+s_{io} \left(i=1,..., m\right) \\ y_{ro}=\sum_{j=1}^{n} y_{rj}\lambda_{j} -s_{ro}\left(r=1,..., s\right) \\ \sum_{j=1}^{n} \lambda_{j}=1 \\ \lambda_{j},s_{io},s_{ro}\geq0, \end{matrix}$$

where $x_{io}$and $y_{ro}$ are the inputs and outputs of DMU_o_ respectively; $s_{io}$and $s_{ro}$ are the input and output inefficiencies (also called ‘slacks’) respectively. (Note: input slacks represent overused inputs, i.e. DMU_o_ could have produced the same amount of output using less input. Output slacks represent output shortfalls, i.e. DMU_o_ could have produced more output given its current input use.); $\lambda_{j}$is a scalar which, when positive, indicates that DMU_j_ has been used as a reference (i.e. benchmark) by DMU_o_; and $R_{i}=\max_{j}\left\{x_{ij}\right\}-\min_{j}\left\{x_{ij}\right\}$, $R_{r}=\max_{j}\left\{y_{rj}\right\}-\min_{j}\left\{y_{rj}\right\}$ represent the ranges in inputs and outputs, respectively, common across *all* DMUs. The ranges act as a ‘data-driven’ weighting scheme, a more objective one compared to methods where the weights are (subjectively) pre-defined by the user. These weights normalize the slacks and make RAM invariant to the units of measurement. The objective function represents the average proportion of the inefficiencies that the ranges show to be possible in each input and output [1]. The constraint $\sum_{j=1}^{n} \lambda_{j}=1$ is the ‘variable returns-to-scale’ specification [2] which ensures that a DMU is only compared to DMUs of similar size.

Model 1 is run $n$ times, once for each farm. When DMU_o_ is efficient all its slacks equal zero as this means that it does not need to further reduce its inputs and increase its outputs to become efficient. In this case RAM inefficiency $\rho^{*}$ in model 1 equals 0, indicating that DMU_o_ is 100% efficient. If DMU_o_ is inefficient, one can identify through the slack values the inputs and desirable outputs contributing the most to its inefficiency. For an inefficient farm any choice of input resulting in $x_{io}>\sum_{j=1}^{n} x_{ij}\lambda_{j}$ means that with some combination of inputs *other* farms (identified by the non-zero $\lambda_{j}$ values) could have improved this input in amount by $s_{io}=x_{io}-\sum_{j=1}^{n} x_{ij}\lambda_{j}$ without worsening any other input or output [3,4]. The same applies for the desirable outputs and their shortfalls $s_{ro}=\sum_{j=1}^{n} y_{rj}\lambda_{j} -y_{ro}$. In either case RAM inefficiency $\rho^{*}$ is greater than 0, indicating that DMU_o_ is inefficient.

Because $\sum_{j=1}^{n} \lambda_{j}=1$ in model 2 it follows that $s_{io}=\sum_{j=1}^{n} \left(x_{io}-x_{ij}\right)\lambda_{j}\leq\sum_{j=1}^{n} R_{i}\lambda_{j}=R_{i}$ and similarly $s_{ro}\leq R_{r}$ and thus $0\leq\rho^{*}\leq1$. Hence, the measure of *inefficiency* $\rho^{*}$ in model 1 can be easily converted to a measure of *efficiency* as follows:

$RAM efficiency=1-\rho^{*}$. (2)

RAM efficiency 2 is bounded by 0 and 1. Unity indicates that the farm under evaluation is efficient while values less than 1 imply that it is inefficient.

Note that RAM carries with it a ranking property, i.e. it can rank inefficient DMUs by their efficiency scores. This is because RAM has two properties that not all other DEA models carry: (i) RAM uses the ranges as a common weighting scheme across *all* DMUs; and (ii) RAM is strongly monotone in the slacks, that is, holding any other inputs and outputs constant, an increase (decrease) in any of its inputs (outputs) will increase the inefficiency score for an inefficient DMU. Properties (i) and (ii) combined make possible the ranking of inefficient DMUs by means of the RAM score.

RAM’s ranking property is very useful for hypothesis testing where two or more groups of DMUs are compared in terms of efficiency scores, such as in Soteriades et al. [4]. This is because the efficiency measures are ordinal but not interval level in data quality [3], hence a convention in the DEA literature is to use non-parametric tests instead of traditional parametric statistics [2,5]. Non-parametric tests that are popular in DEA studies (e.g. Kolmogorov-Smirnov test; Kruskal-Wallis test; Mann-Whitney test; Spearman’s and Kendall’s rank correlation coefficients) are based on the ranks of the observations instead of the observations themselves and therefore RAM’s ranking property is very beneficial in this case.

Nevertheless, reporting the scores’ ranks rather than the actual scores might not be easily communicated in many cases and so several studies also report summary statistics on the scores [4,6,7]. Although summary statistics are in conflict with the data quality of the efficiency scores (see previous paragraph), Bogetoft and Otto [5] argue for a relaxation of the ‘theoretical niceties’ (p.196) characterizing DEA so as to expand the method’s applicability. They also provide several examples of studies having successfully done so by feeding the DEA scores into regression models. This is also done in this study as the DEA scores were fed into PLS-SEM models.

# References

1. Cooper WW, Park KS, Pastor JT. RAM: a range adjusted measure of inefficiency for use with additive models, and relations to other models and measures in DEA. J Prod Anal. 1999;11: 5-42.

2. Cooper WW, Seiford LM, Tone K. Data envelopment analysis: a comprehensive text with models, applications, references and DEA-Solver software. 2nd ed. Springer Science+Business Media, LLC; 2007.

3. Brockett PL, Cooper WW, Golden LL, Rousseau JJ, Wang YY. Evaluating solvency versus efficiency performance and different forms of organization and marketing in US property - liability insurance companies. Eur J Oper Res. 2004; 154: 492-514.

4. Soteriades AD, Faverdin P, Moreau S, Charroin T, Blanchard M, Stott AW. An approach to holistically assess (dairy) farm eco-efficiency by combining life cycle analysis with data envelopment analysis models and methodologies. Animal 2016;10: 1899-1910.

5. Bogetoft P, Otto L. Benchmarking with DEA, SFA, and R. Springer; 2011.

6. Leverty JT, Grace MF. Issues in measuring the efficiency of property-liability insurers. 2008; http://www3.imperial.ac.uk/pls/portallive/docs/1/48217697.PDF

7. Sueyoshi T, Goto M. Should the US clean air act include CO2 emission control? Examination by data envelopment analysis. Energy Policy 2010;38: 5902-5911.
