# Supplementary material for: The Relationship of Dairy Farm Eco-Efficiency with Intensification and Self-Sufficiency. Evidence from the French Dairy Sector Using Life Cycle Analysis, Data Envelopment Analysis and Partial Least Squares Structural Equation Modelling
Source: PLoS One. 2016 Nov 10;11(11):e0166445. doi: 10.1371/journal.pone.0166445 (PMC5104379; doi:10.1371/journal.pone.0166445)
Supplement: S2 Appendix — (DOCX) [file pone.0166445.s002.docx]

# S2 Appendix. Equations for the PLS-SEM model in Fig 1

## Measurement model

Assume that there are *j* latent variables and that each one is represented by *k* manifest variables. Each manifest variable is related to its latent variable by a simple regression:

*MV_jk_* = *λ_0jk_* + *λ_jk_ LV_j_* + *ε_jk_*, (1)

where *MV_jk_* represents the *k*th manifest variable of the *j*th latent variable (*LV_j_*); *λ_0jk_* is the intercept term; *λ_jk_* are the loadings; and *ε_jk_* are the error terms accounting for the residuals. The only hypothesis made on model 1 is the *predictor specification* condition:

*E*(*MV_jk_* | *LV_j_*) = *λ_0jk_* + *λ_jk_ LV_j_*, (2)

implying that that *ε_jk_* has a zero mean and is uncorrelated with *LV_j_*. Based on model 1, the measurement model in Fig 1 can be expressed as follows:

(Milk/cow) = *λ*_011_ + *λ*_11_ *INTENS-A* + *ε*_11_

(Meat/LU) = *λ*_012_ + *λ*_12_ *INTENS-A* + *ε*_12_

(Concentrate/LU) = *λ*_013_ + *λ*_13_ *INTENS-A* + *ε*_13_

(N/on-farm ha) = *λ*_021_ + *λ*_21_ *INTENS-F* + *ε*_21_

(P/on-farm ha) = *λ*_022_ + *λ*_22_ *INTENS-F* + *ε*_22_

(Stocking density) = *λ*_023_ + *λ*_23_ *INTENS-F* + *ε*_23_

(Maize/forage ha) = *λ*_024_ + *λ*_24_ *INTENS-F* + *ε*_24_

(Economic) = *λ*_031_ + *λ*_31_ *SELF* + *ε*_31_

(Energy) = *λ*_032_ + *λ*_32_ *SELF* + *ε*_32_

(Feed) = *λ*_033_ + *λ*_33_ *SELF* + *ε*_33_

(Land) = *λ*_034_ + *λ*_34_ *SELF* + *ε*_34_

(DEA eco-efficiency) = *λ*_041_ + *λ*_41_ *ECO* + *ε*_41_

Note that the raw data must be standardized (mean = 0, variance = 1) before being fed into the model above because doing so allows the PLS-SEM model to calculate standardized coefficients between -1 and +1 for every relationship in the measurement and structural models [1].

## Structural model

The causality model in Fig 1 leads to linear equations relating the predictor latent variables (*INTENS-A*, *INTENS-F* and *SELF*) with the response latent variable (*ECO*):

*ECO* = *β*_0_ + *β*_1_ *INTENS-A* + *β*_2_ *INTENS-F* + *β*_3_ *SELF* + ε, (3)

where; *β*_0­_ is the intercept term; *β*_1_, *β*_2_ and *β*_3_ are the path coefficients; and ε is the error term accounting for the residuals. Note that the *predictor specification* condition also applies to structural model 3. See [2,3].

# References

1. Hair JF, Hult GT, Ringle CM, Sarstedt M. A primer on partial least squares structural equation modelling (PLS-SEM). SAGE Publications Inc; 2014.

2. Sanchez G. PLS Path Modeling with R. Berkeley: Trowchez Editions; 2013.

3. Tenenhaus M, Vinzi VE, Chatelin YM, Lauro C. PLS path modeling. Comput Stat Data An. 2005;48: 159-205.
