# Supplementary material for: The Relationship of Dairy Farm Eco-Efficiency with Intensification and Self-Sufficiency. Evidence from the French Dairy Sector Using Life Cycle Analysis, Data Envelopment Analysis and Partial Least Squares Structural Equation Modelling
Source: PLoS One. 2016 Nov 10;11(11):e0166445. doi: 10.1371/journal.pone.0166445 (PMC5104379; doi:10.1371/journal.pone.0166445)
Supplement: S3 Appendix — (DOCX) [file pone.0166445.s003.docx]

# S3 Appendix. Equations for PLS-SEM-ALL and PLS-SEM-AF

## PLS-SEM-ALL

The measurement model for PLS-SEM-ALL (Fig 3 in main text) is as follows:

(Milk/cow) = *λ*_011_ + *λ*_11_ *INTENS-ALL* + *ε*_11_

(Concentrate/LU) = *λ*_012_ + *λ*_12_ *INTENS-ALL* + *ε*_12_

(N/on-farm ha) = *λ*_013_ + *λ*_13_ *INTENS-ALL* + *ε*_13_

(Maize/forage ha) = *λ*_014_ + *λ*_14_ *INTENS-ALL* + *ε*_14_

(Energy-IC) = *λ*_015_ + *λ*_15_ *INTENS-ALL* + *ε*_15_

(Feed-IC) = *λ*_016_ + *λ*_16_ *INTENS-ALL* + *ε*_16_

(DEA eco-efficiency) = *λ*_021_ + *λ*_21_ *ECO* + *ε*_21_

The structural model for PLS-SEM-ALL is as follows:

*ECO* = *β*_0_ + *β*_1_ *INTENS-ALL* + ε

## PLS-SEM-AF

The measurement model for PLS-SEM-AF (Fig 4 in main text) is as follows:

(Milk/cow) = *λ*_011_ + *λ*_11_ *INTENS-AF* + *ε*_11_

(Concentrate/LU) = *λ*_012_ + *λ*_12_ *INTENS-AF* + *ε*_12_

(N/on-farm ha) = *λ*_013_ + *λ*_13_ *INTENS-AF* + *ε*_13_

(Maize/forage ha) = *λ*_014_ + *λ*_14_ *INTENS-AF* + *ε*_14_

(DEA eco-efficiency) = *λ*_021_ + *λ*_21_ *ECO* + *ε*_21_

The structural model for PLS-SEM-AF is as follows:

*ECO* = *β*_0_ + *β*_1_ *INTENS-AF* + ε

Note that the raw data must be standardized (mean = 0, variance = 1) before being fed into the models above because doing so allows the PLS-SEM model to calculate standardized coefficients between -1 and +1 for every relationship in the measurement and structural models [1].

# References

1. Hair JF, Hult GT, Ringle CM, Sarstedt M. A primer on partial least squares structural equation modelling (PLS-SEM). SAGE Publications Inc; 2014.
