# Supplementary material for: The Relationship of Dairy Farm Eco-Efficiency with Intensification and Self-Sufficiency. Evidence from the French Dairy Sector Using Life Cycle Analysis, Data Envelopment Analysis and Partial Least Squares Structural Equation Modelling
Source: PLoS One. 2016 Nov 10;11(11):e0166445. doi: 10.1371/journal.pone.0166445 (PMC5104379; doi:10.1371/journal.pone.0166445)
Supplement: S3 Table — INTENS-AF: animal and farm-level intensification; ECO: eco-efficiency; CI: confidence interval; AVE: average variance extracted; LU: livestock unit; DEA: data envelopment analysis; N/A: not applicable. Rules of thumb [30,42,45,52]: loadings, Cronbach’s alpha and Dillon-Goldstein’s rho should be at least 0.70. Communalities (squared loadings) and AVE should be at least 0.50. First and second eigenvalues should be above and below 1 respectively. Note: ECO is a single-item construct so assessment criteria above do not apply. (DOCX) [file pone.0166445.s007.docx]

**S3 Table. Indicator reliability, internal consistency reliability and convergent validity of the measurement model for PLS-SEM-AF for Continental Specialized Systems (CSS).**

| **Constructs** | **Manifest variables** | **Indicator reliability** | | **Internal consistency reliability** | | | | **Convergent validity** |
| --- | --- | --- | --- | --- | --- | --- | --- | --- |
|  |  | **Loadings (95% CI)** | **Communalities** | **Cronbach’s *alpha*** | **Dillon-Goldstein’s *rho*** | **Correlation matrix** | | **AVE** |
|  |  |  |  |  |  | **1st eigenvalue** | **2nd eigenvalue** |  |
| *INTENS-AF* | Milk/cow | 0.89 (0.59, 0.94) | 0.79 | 0.86 | 0.92 | 2.36 | 0.41 | 0.78 |
|  | Concentrate/LU | 0.91 (0.81, 0.99) | 0.83 |  |  |  |  |  |
|  | Maize/forage ha | 0.84 (0.46, 0.92) | 0.71 |  |  |  |  |  |
| *ECO* | DEA eco-efficiency | N/A | N/A | N/A | N/A | N/A | N/A | N/A |

*INTENS-AF*: animal and farm-level intensification; *ECO*: eco-efficiency; CI: confidence interval; AVE: average variance extracted; LU: livestock unit; DEA: data envelopment analysis; N/A: not applicable. Rules of thumb [30,42,45,52]: loadings, Cronbach’s *alpha* and Dillon-Goldstein’s *rho* should be at least 0.70. Communalities (squared loadings) and AVE should be at least 0.50. First and second eigenvalues should be above and below 1 respectively. Note: *ECO* is a single-item construct so assessment criteria above do not apply.
